# Genetic Diversity and Population Structure of *Squalus* cf. *mitsukurii* (Chondrichthyes: Squalidae) Revealed by ddRAD Sequencing

**DOI:** 10.1002/age.70096

**Published:** 2026-03-31

**Authors:** Aisni Mayumi Corrêa de Lima Adachi, Ailton Amarante Ariza, Giovana da Silva Ribeiro, Pollyana Christine Gomes Roque, Matheus Marcos Rotundo, Marcelo Vianna, Gabriela Delpiani, Sergio Matias Delpiani, Juan Martín Díaz de Astarloa, Claudio Oliveira, Fausto Foresti, Vanessa Paes da Cruz

**Affiliations:** ^1^ Laboratório de Biologia e Genética de Peixes, Instituto de Biociências de Botucatu Universidade Estadual Paulista Botucatu Brazil; ^2^ Laboratório de Oceanografia Pesquisa Universidade Federal Rural de Pernambuco Recife Brazil; ^3^ Acervo Zoológico da Universidade Santa Cecília (AZUSC) Universidade Santa Cecília Santos Brazil; ^4^ Laboratório de Biologia e Tecnologia Pesqueira Universidade Federal Do Rio de Janeiro Rio de Janeiro Brazil; ^5^ Grupo de Biotaxonomía Morfológica y Molecular de Peces (BIMOPE) Instituto de Investigaciones Marinas y Costeras (IIMyC, CONICET‐UNMdP) Mar del Plata Argentina; ^6^ Consejo Nacional de Investigaciones Científicas y Técnicas (CONICET) Buenos Aires Argentina; ^7^ Laboratorio de Ecología, Fisiología y Evolución de Organismos Acuaticos (LEFyE) Centro Austral de Investigaciones Científicas (CADIC‐CONICET) Ushuaia Argentina

**Keywords:** population genomics, shark conservation, shortspine spurdog, single nucleotide polymorphisms (SNPs)

## Abstract

Assessing genetic structure across ocean basins is essential to understand connectivity and guide conservation in data‐deficient open‐water sharks. In this study, we examined the population genomics of *Squalus* cf. *mitsukurii* by analyzing tissue samples collected from two distant regions: California, USA (Pacific Ocean) and Pernambuco, Brazil (Southwestern Atlantic Ocean). Using double‐digest restriction site‐associated DNA sequencing (ddRADseq), we generated 33 480 939 raw reads across 28 individuals and retained 1118 high‐quality single nucleotide polymorphisms (SNPs) after stringent filtering. Genetic diversity was moderate (He = 0.211–0.244), with low inbreeding levels (*F*_is_ = −0.005–0.106). DAPC and pairwise *F*_st_ analyses revealed moderate genetic structure, indicating limited gene flow between the sampled populations. This study provides the first genomic evidence of population differentiation in *S.* cf. *mitsukurii* across these regions. Our findings underscore the importance of genome‐wide data and informing regionally conservation strategies, especially for exploited species commonly misidentified in commercial landings and lacking population management.

## 1 Introduction

Elasmobranchs (rays and sharks) represent the second most threatened group of vertebrates, with approximately 37% of species categorized under some level of extinction risk (Dulvy et al. 2021; IUCN 2025). This vulnerability stems from their life history traits, including slow growth, low fecundity, and late sexual maturity (Cortés 2000; Ebert et al. 2021; Whitenack et al. 2022). Moreover, elasmobranchs are particularly susceptible to anthropogenic threats, such as overfishing, largely driven by the global fin trade (Yano et al. 2022; Sherman et al. 2023; Osuka et al. 2025). Although the sale of meat from endangered species is prohibited in many regions (Feitosa et al. 2018; Alvarenga et al. 2021, 2024) several deepwater species are still exploited for their liver oil in the international squalene trade (Finucci, Pacoureau, et al. 2024; Finucci, Rigby, et al. 2024). These combined pressures have contributed to population declines and heightened extinction risks (Dulvy et al. 2021; Pacoureau et al. 2021; Sherman et al. 2023; Mejía et al. 2025).

The genus *Squalus* Linnaeus, 1758 (Chondrichthyes, Squalidae), commonly known as dogfish, includes small sharks reaching up to 120 cm in total length, occurring at depths ranging from 100 to 500 m (Compagno 1984, 2008; Ebert et al. 2021). The genus comprises 41 recognized species (Van Der Laan and Fricke 2023) with a wide distribution across the Atlantic, Pacific, and Indian Oceans (Last et al. 2006; Daly‐Engel et al. 2018; Pfleger et al. 2018; Ziadi‐Künzli et al. 2020; Ebert et al. 2021; Andreoli et al. 2025). Due to morphological similarities among species, taxonomic identification is challenging and has led to the classification of species into taxonomic complexes, namely the *acanthias*, *megalops/cubensis*, and *blainvillei/mitsukurii* (Viana et al. 2016; Veríssimo et al. 2017; Bellodi et al. 2018; Pfleger et al. 2018; Ebert et al. 2021; Ferrari et al. 2021; Adachi et al. 2023).

The species *Squalus mitsukurii* , originally described from Misaki, Kanagawa, Japan (Jordan and Snyder, 1903), is currently considered to be restricted to the Pacific Ocean. However, its occurrence in the Atlantic has been debated (Bonello et al. 2016; Pfleger et al. 2018; Ariza et al. 2022; Andreoli et al. 2025). Recent studies indicate that many individuals previously identified as *S. mitsukurii* may belong to other species, such as *S. hawaiiensis* (Daly‐Engel et al. 2018), *S. clarkae* (Pfleger et al. 2018), and *S. shiraii* (Viana and De Carvalho 2020), which often exhibit high degrees of endemism. Nevertheless, morphologically similar individuals from the *blainvillei/mitsukurii* complex continue to be reported across the Atlantic, Indian, and Pacific Oceans, suggesting either the existence of widely distributed species or the presence of undescribed species within the *S. mitsukurii* complex (Viana et al. 2016; Pfleger et al. 2018; Finucci et al. 2020; Ziadi‐Künzli et al. 2020; Andreoli et al. 2025). In this context, individuals analyzed in the present study were sampled from California (eastern Pacific) and northeastern Brazil (western Atlantic), representing geographically distant regions where members of the blainvillei/mitsukurii complex have been reported.

*Squalus mitsukurii* exhibits a continuous reproductive cycle without clear seasonality, although a peak in mating activity has been reported between February and August (Furumitsu et al. 2012). Observations along the southern coast of Brazil also indicate reproductive activity throughout the year, during fall and winter (Lourenço et al. 2025). Females reach sexual maturity at a larger size than males, and fecundity ranges from one to five embryos per gestation (Graham 2005; Furumitsu et al. 2012). These life history traits, including delayed maturity, low fecundity, and possibly extended reproductive cycles, reduce the species' resilience to fishing pressures compared to other marine organisms (Reynolds et al. 2005; Field et al. 2009; Dulvy et al. 2017, 2021, 2024). As a result, *S. mitsukurii* is currently listed as Endangered (EN) by the IUCN (International Union for Conservation of Nature) Red List of Threatened Species (Finucci et al. 2020). The species is commonly caught as bycatch in mixed‐species demersal trawl fisheries, which has led to population depletion in heavily fished areas. However, some populations may persist in less exploited habitats, potentially offering resilience to overfishing (Gallagher et al. 2012; Shipley et al. 2023; Moro et al. 2025).

The absence of species‐specific management strategies exacerbates conservation challenges and hinders accurate assessment of fishing impacts on the long‐term survival of the species (Finucci, Pacoureau, et al. 2024; Finucci, Rigby, et al. 2024). Understanding the population structure of a species and the factors driving its dispersal is crucial for evaluating conservation status and mitigating the risk of local extinction (Allendorf 2017; Frankham et al. 2017; Jorgensen et al. 2022; Fricke et al. 2025).

In this context, identifying genetically distinct populations becomes essential for conservation planning. These populations may require tailored management actions due to differences in ecological, genetic, or demographic characteristics (Moritz 1994; Funk et al. 2012). Recognizing these distinct populations within widely distributed or taxonomically complex species like *Squalus* cf. *mitsukurii* enables more accurate assessments of conservation priorities and the implementation of region‐specific strategies.

Double‐digest restriction site‐associated DNA sequencing (ddRAD) is a reduced‐representation genomic approach that enables the generation of thousands of genome‐wide SNPs without the need for a reference genome, making it particularly suitable for non‐model organisms such as elasmobranchs (Kumar and Kocour 2017; Allendorf et al. 2022).

Genome‐wide SNP datasets generated via ddRADseq are increasingly important for conservation because their high genomic density provides greater statistical power to detect fine‐scale population structure and connectivity (Clark et al. 2025; Meyers et al. 2025). This is especially relevant for elasmobranchs, as such resolution enables the identification of cryptic barriers to gene flow and informs more effective management strategies, even in species with high dispersal potential.

To date, no reference genome or extensive genomic resources are available for *Squalus* cf. *mitsukurii*, for which the use of “cf.” reflects ongoing taxonomic uncertainty within the *Squalus mitsukurii* species complex and acknowledges unresolved species boundaries reported for this group, reinforcing the utility of ddRAD for assessing population genetic structure and diversity in this species. Therefore, the aim of this study was to assess the genetic diversity of *Squalus* cf. *mitsukurii* in two distinct coastal regions, in Brazil (Pernambuco) and the United States (California), using ddRADseq.

## 2 Material and Methods

### 2.1 Samples Collection

Samples from the muscle tissue of 28 *Squalus* cf. *mitsukurii* individuals, captured in two coastal regions: California, United States of America (USA, *n* = 11), and Pernambuco, Brazil (BRA, *n* = 17) were used. These samples are deposited in the tissue collection of the Laboratory of Fish Biology and Genetics (LBP) at UNESP, Botucatu, São Paulo, Brazil. Specimens were identified based on external morphological characters following regional taxonomic keys for *Squalus* species, and identifications were confirmed by experienced elasmobranch taxonomists. Photographic records were obtained for representative individuals from each sampling location, and molecular identification was conducted as described by Ariza et al. (2022). Tissue fragments were preserved in 95% ethanol and stored at −20°C. All sample collections were conducted in accordance with Brazilian regulations (SISBIO protocol 13843‐1 and 39978‐4) and approved by the Animal Ethics Committee (protocol number 3362).

### 2.2 Extraction of DNA and ddRAD Library Preparation

Genomic DNA was extracted from preserved muscle tissue fragments using the Wizard Genomic DNA Purification kit (Promega, Madison, WI, USA), following the manufacturer's instructions. DNA concentration was quantified using a Qubit 4.0 Fluorometer (Invitrogen). The DNA was then standardized for the digestion reaction to a final volume of 34 μL, with a concentration of 200 ng per sample (5.88 ng/μL).

The preparation of the library was performed using the ddRADseq technique following the protocol described by Peterson et al. (2012) and adapted by Campos et al. (2017). For the enzymatic digestion, 1 μL of each restriction enzyme was used: EcoRI (20 U/μL) and MspI (10 U/μL). The digestion reaction was prepared in a total volume of 40 μL, with 4 μL of TANGO buffer (Cut Smart). The reaction was incubated at 37°C for 3 h. Following enzymatic digestion, samples were purified using the Agencourt AMPure XP beads kit (Beckman Coulter, USA), according to the manufacturer's protocol. This purification step aimed to remove residual enzymes and eliminate DNA fragments smaller than 100 base pairs and larger than 500 base pairs.

DNA concentration in each sample was then quantified using a Qubit 4.0 Fluorometer (Invitrogen) to proceed with the adapter ligation step. Then, a pair of hybridized adapters—P1 (3nM—EcoRI) and P2 (6 nM—MspI) complementary to each enzyme's recognition site were added. Adapter ligation reaction was performed using 31.5 μL of the digestion product, 4 μL of 10× T4 DNA Ligase Reaction Buffer (Promega), and 2 μL of each adapter, resulting in a final volume of 40 μL. The adapter ligation was incubated at 23°C for 30 min, followed by 63°C for 10 min, and then held at 63°C for 90 s. After that, the temperature was reduced by 2°C every 90 s until reaching 23°C. Following this thermal cycling, the purification process was repeated using the Agencourt AMPure XP beads kit (Beckman Coulter, USA).

Following adapter ligation, indexing reactions were performed by adding the Nextera Index Primers (Illumina, San Diego USA) S500 and N700 (Nextera DNA CD Indexes—96 indexes, 96 samples). Each sample received a unique combination of index primers, allowing for individual identification. Indexing was carried out using the Nextera DNA Sample Preparation Kit (Illumina), targeting the DNA fragments ligated to the adapters. The indexing reaction included 15 μL of the ligation product, 25 μL of Phusion High‐Fidelity PCR Master Mix (Thermo Scientific), 5 μL of each index primer (S500 and N700), resulting in a final volume of 50 μL. Afterward, PCR amplification was carried out using a thermocycler with the following conditions: an initial incubation at 72°C for 3 min, followed by 16 cycles at 95°C for 30 s, 55°C for 30 s and 72°C for 30 s; and a final extension at 72°C for 5 min, with a final hold at 4°C indefinitely. After amplification, the samples underwent a new purification step.

DNA was quantified using the Qubit 4.0 Fluorometer (Invitrogen) and standardized to a final concentration of 10 ng/μL, and then pooled and purified using the Agencourt AMPure XP beads (Beckman Coulter, USA). The purified product was quantified using the Qubit 4.0 Fluorometer (Invitrogen), and subsequently, a 1% agarose gel electrophoresis was performed to visualize the fragments. DNA fragments of interest, ranging from 300 to 500 bp, were selected and purified using the Wizard SV Gel and PCR Clean‐Up System kit (Promega, USA). The final library was quantified by real‐time PCR (qPCR), normalized, and sequenced as 150 bp single‐end reads at the Instituto de Biotecnologia (IBETEC), Universidade Estadual Paulista (UNESP), Botucatu, Brazil, using the Illumina NextSeq 550 next‐generation sequencing (NGS) platform.

### 2.3 Bioinformatics Analysis

Multiple steps were performed during data analysis. First, the quality of the reads (raw data) and the presence of adapter sequences were assessed using the software FastQC v0.12.0 (Andrews 2010) and MultiQC v1.28 (Ewels et al. 2016). Afterward, low quality reads (Quality score < 20) and adapter sequences were removed, and the remaining reads were trimmed and standardized to 143 bp in length using the software Trimmomatic v0.32 (Bolger et al. 2014). In addition, an *in silico* digestion was performed following Driller et al. (2020) to remove reads containing internal restriction enzyme recognition sites.

After the standardization, downstream SNPs analysis was conducted using the Stacks v2 pipeline (Catchen et al. 2013). SNP analyses were conducted according to the parameter recommendations proposed by Paris et al. (2017), using *M* = 2, *m* = 3, and *n* = 1. Then, read alignment was performed using Bowtie2 v2.2.4 (Langmead and Salzberg 2012) software. SNP filtering was carried out using the *populations* module of the Stacks pipeline, applying three filtering criteria: (1) SNPs were required to be present in at least 70% of individuals per population (*r* = 0.70); (2) SNPs with a minor allele frequency (MAF) below 0.05 were excluded; and (3) SNPs with a maximum observed heterozygosity value greater than 0.65 were removed (parameter ‐‐max‐obs‐het = 0.65). To reduce potential linkage among loci, only one SNP per RAD locus was retained for downstream analyses.

The indices of private alleles (Ap) were initially calculated using the populations module in Stacks. Observed heterozygosity (Ho), expected heterozygosity (He), inbreeding coefficient (*F*_is_) and pairwise *F*_ST_ values were estimated using the software Arlequin v3.5.2.2 (Excoffier and Lischer 2010).

Then, Discriminant Analysis of Principal Components (DAPC) (Jombart et al. 2010) was performed to identify genetic clusters using the adegenet R package (Jombart 2008; Jombart and Ahmed 2011). Population structure analysis was further evaluated using the software STRUCTURE (Pritchard et al. 2000) to assess sample partitioning into genetic clusters. *K*‐value estimations ranged from *K* = 1 to *K* = 4, with 1 000 000 MCMC repetitions and a 10% burn‐in across 10 independent runs per K, assuming the correlated admixture model and allele frequency correlation. The most likely number of genetic clusters (K) was determined by the Evanno method (Evanno et al. 2005) and Puechmaille methods (Puechmaille 2016), implemented via the Structure Selector tool (Li and Liu 2018). Results were averaged and visualized using the CLUMPAK software (Kopelman et al. 2015). Although both DAPC and STRUCTURE assess population genetic they are complementary approaches: DAPC is a multivariate, non‐model‐based approach that does not assume Hardy–Weinberg equilibrium, whereas STRUCTURE relies on Bayesian clustering under explicit population genetic assumptions.

## 3 Results

The ddRAD library resulted in a total of 33 480 939 raw reads, with reads counts per individual of *Squalus* cf. *mitsukurii* ranging between 426 695 and 1 362 078. After quality filtering (*Q* < 20), a total of 26 795 041 high‐quality reads were retained. All reads were trimmed to 143 bp for downstream analysis to ensure consistency. The dataset included between 426 062 and 1 357 950 reads per individual (Data S1).

**FIGURE 1:**
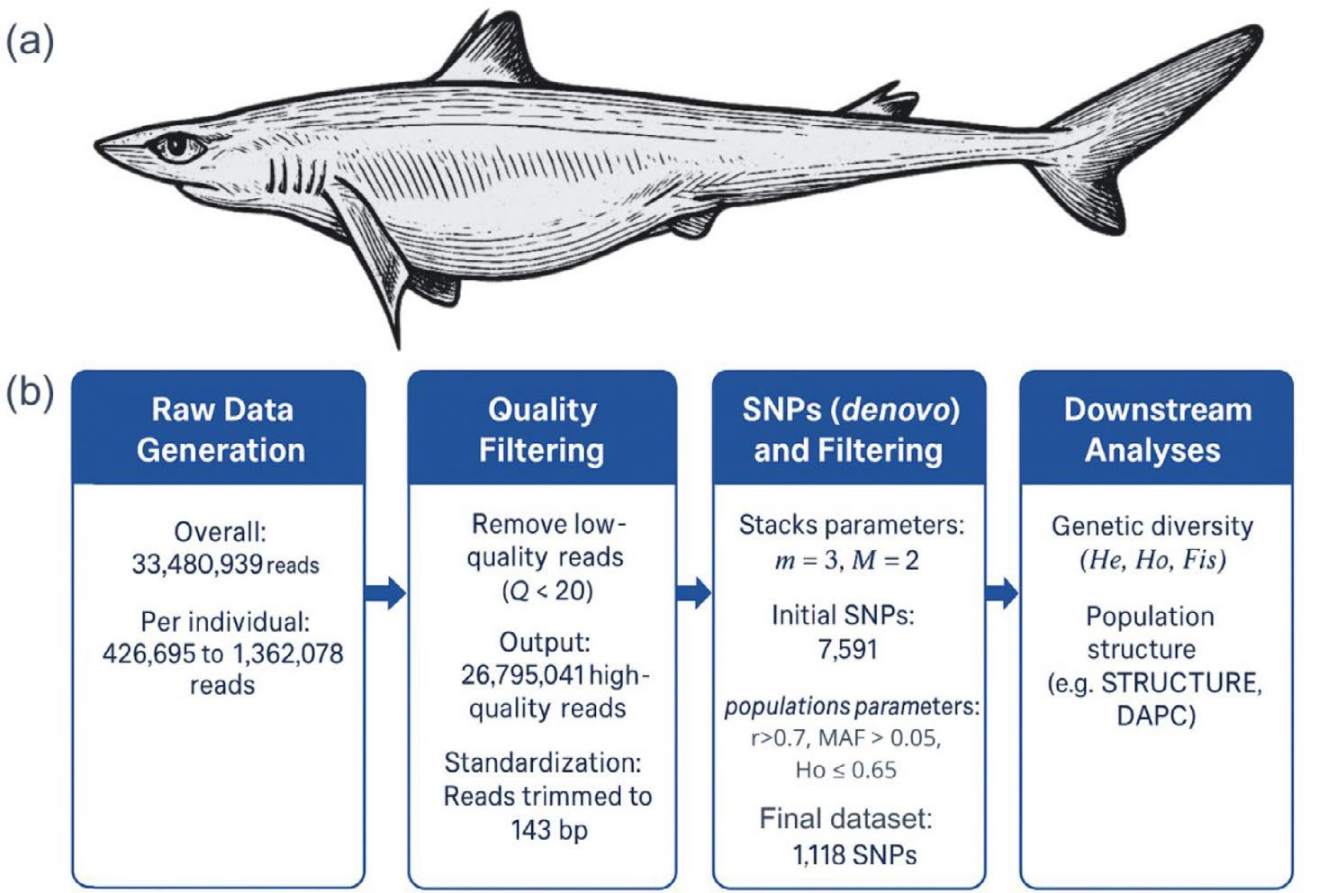
(a) Graphic representation of *Squalus* cf. *mitsukurii* generated by artificial intelligence, for illustrative purposes only. (b) Method workflow of bioinformatics.

Using de novo assembly in STACKS, we identified an initial set of 7591 SNPs across all individuals. Effective per‐sample sequencing depth averaged 2.0× (range: 2.0–2.9×). Stringent filtering resulted in a final dataset comprising a total of 1118 SNPs, yielding a high‐quality SNP dataset with concordant patterns recovered across downstream analyses, which were subsequently used for further analysis (Figure 1).

**FIGURE 2:**
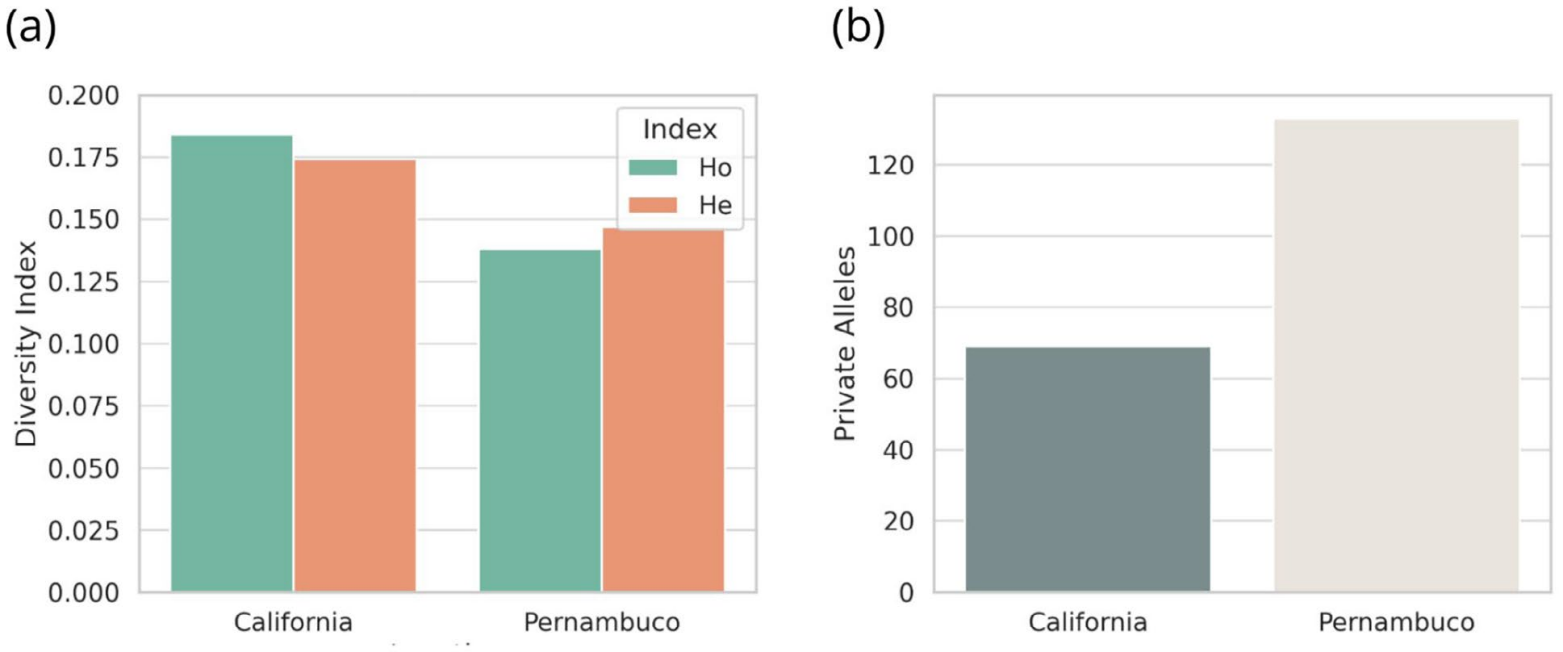
Genetic diversity parameters estimated for *Squalus* cf. *mitsukurii* populations sampled off the coasts of California (USA; *n* = 11) and Pernambuco (BRA; *n* = 17), based on 1118 SNPs. (a) heterozygosity observed (Ho) and expected (He); (b) number of private alleles.

Observed heterozygosity values were relatively higher in one population; however, these results should be interpreted as indicative patterns rather than definitive measures of overall genetic diversity. Genetic diversity metrics revealed differences between the two sampling sites. In terms of heterozygosity, California exhibited a pattern where observed homozygosity (Ho = 0.184) exceeded expected heterozygosity (He = 0.174) (Figure 2a). Conversely, in Pernambuco, the opposite pattern was observed, with Ho (0.138) lower than He (0.147) (Figure 2a). These values suggest that the California population harbors greater genetic variability, while Pernambuco shows comparatively reduced variability. A total of 69 private alleles were identified in California, whereas 133 private alleles were detected in Pernambuco, indicating a greater number of unique variants in the latter population (Figure 2b). The inbreeding coefficient (*F*_is_) was negative for both locations, with values of −0.0472 in California and −0.0567 in Pernambuco, suggesting no evidence of inbreeding based on Fis estimates; however, given the number of loci analyzed, this interpretation should be made with caution.

**FIGURE 3:**
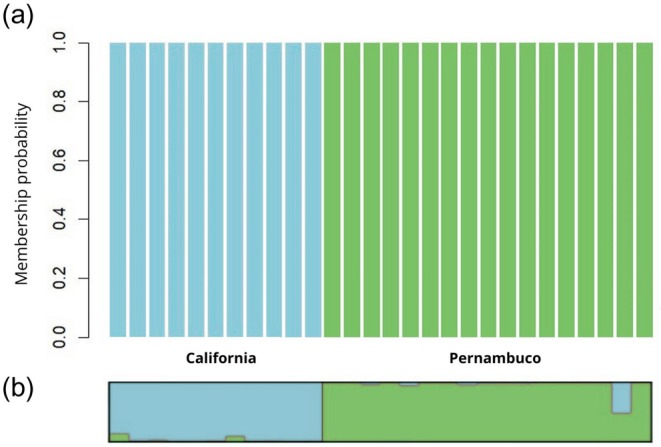
Genetic structure of *Squalus* cf. *mitsukurii* based on 1118 SNPs from individuals sampled in California (USA, blue color) and Pernambuco (Brazil, green color). (a) Discriminant Analysis of Principal Components (DAPC) plot showing the genetic differentiation between the two regional groups. Each vertical bar represents a single individual (b) Genetic clustering results from STRUCTURE, visualized using CLUMPAK. The plot indicates the most likely number of genetic clusters (*K* = 2).

Genetic differentiation between the two sampling sites was supported by the pairwise fixation index (Fst), which revealed a statistically significant value of 0.246 between California and Pernambuco. This result indicates a high level of genetic structure and limited connectivity between the two populations. Complementary evidence was provided by the Discriminant Analysis of Principal Components (DAPC), which identified the presence of two distinct genetic clusters, clearly separating individuals according to their geographic origin (Figure 3a).

The STRUCTURE analysis identified *K* = 2 as the most likely number of genetic clusters, as determined by Structure Selector based on model fit criteria (Figure 3b, Data S2). The resulting admixture plot showed that while individuals were predominantly assigned to one of the two clusters, several exhibited mixed ancestry, with varying proportions of genetic membership shared between the two groups.

## 4 Discussion

In this study, we conducted the first assessment of genomic diversity in *Squalus* cf. *mitsukurii*, using 1118 SNP markers to evaluate the genetic diversity and population structure between two geographically distant populations from the Pacific (California, USA) and Atlantic (Pernambuco, BRA) coasts. The analyses revealed a marked genetic differentiation between these populations, suggesting restricted gene flow across these oceans and indicating that they are distinct populations.

Genetic diversity values in *S*. cf. *mitsukurii* appear to be comparatively low when contrasted with other species of the genus. For instance, *Squalus albicaudus* has been reported to exhibit observed heterozygosity ranging from 0.230 to 0.423, and expected heterozygosity from 0.212 to 0.377, indicating relatively higher levels of genomic variability (Adachi et al. 2023). In contrast, *S*. cf. *mitsukurii* populations from both Atlantic and Pacific regions display markedly reduced heterozygosity, with values below 20%, suggesting limited genetic diversity.

Although heterozygosity levels were generally low, the inbreeding coefficient was negative in both populations, suggesting an absence of inbreeding. This pattern is frequently observed in open‐sea elasmobranchs, where reduced genetic diversity does not necessarily coincide with elevated inbreeding (Duchatelet et al. 2020; Leone et al. 2024). Similar trends have been reported in species such as *Somniosus pacificus* and *S. microcephalus* , both of which consistently exhibit heterozygosity levels below 15% and negative *F*_is_ values (Timm et al. 2023). These patterns are often interpreted as indicators of historical demographic stability or admixture, rather than recent inbreeding, even in populations with limited genetic variability. Notably, both species are currently listed under conservation concern by the IUCN, with *Somniosus pacificus* categorized as Near Threatened (Rigby et al. 2021) and *Somniosus microcephalus* as Vulnerable (Kulka et al. 2020). These cases highlight how *K* reproductive strategy, combined with open‐sea isolation and historical bottlenecks, can shape patterns of genetic diversity and population structure without necessarily producing inbreeding effects (Di Crescenzo et al. 2022; Matta et al. 2024).

Despite the lower genetic diversity observed in most elasmobranchs, including *S. mitsukurii* (Daly‐Engel et al. 2018), our study reveals a contrasting pattern. In the case of *Squalus* cf. *mitsukurii*, higher genetic diversity was observed in the California region. Even so, ongoing anthropogenic pressures such as pollution, overfishing, and climate change could threaten this stability, including the long‐term reduction of genetic diversity and alterations in population structures, similar to those observed in other marine species (Allendorf et al. 2008; Dulvy et al. 2021; Hohenlohe et al. 2021; Yuan et al. 2023).

The population structure estimates obtained in this study for *Squalus* cf. *mitsukurii* suggest a higher genetic differentiation between the two analyzed regions. Both STRUCTURE and DAPC analysis indicate that the two regions demonstrate clear genetic structuring, likely driven by the considerable geographic distance separating the two oceanic regions. Although clear genetic structuring was detected, the relatively limited sample size suggests cautious interpretation of these results. Future studies with broader sampling across the species' range will help refine estimates of genetic diversity and population connectivity.

In addition to spatial separation, ecological and behavioral factors may further contribute to the formation and maintenance of distinct genetic stocks. Although no direct studies have investigated the migratory behavior of *S. mitsukurii* , existing evidence indicates that its reproductive cycle is seasonal, which may imply migratory movements associated with breeding or responses to environmental changes (Lourenço et al. 2025). The two genetically distinct stocks observed in this study may therefore result from limited gene flow between regions, potentially driven by behavioral patterns such as site fidelity or reproduction linked movements that do not involve long distance migration across ocean basins. Consistent with this, a review by Kottillil et al. (2023), highlights that patterns of phylogeography and population structure in sharks and rays reveal that most species exhibit genetic structuring either within or across ocean basins (Devloo‐Delva et al. 2023). For example, the pelagic sand tiger shark, *Carcharias taurus* , displays genetic structuring within the Atlantic basin, likely due to the barrier effects of warm equatorial currents combined with strong site fidelity to breeding areas (Ahonen et al. 2009). Similarly, the blacktip reef shark, *Carcharhinus melanopterus* , exhibits population structuring observed in the Pacific basin, influenced by deep oceanic waters and a tendency toward philopatry (Vignaud et al. 2014). Additionally, the species *Carcharhinus longimanus* , shows evidence of global population genetic structure, with mitochondrial and nuclear DNA markers revealing differentiation between Western Atlantic and Indo‐Pacific populations (Ruck et al. 2024).

Assessing genetic diversity and population structure in elasmobranchs is essential for preserving evolutionary potential in fragmented populations increasingly threatened by anthropogenic pressures (Domingues et al. 2018; Ziadi‐Künzli et al. 2020; Theissinger et al. 2023). In this context, the genetic differentiation observed between *Squalus* cf. *mitsukurii* populations highlights the need for conservation strategies that are regionally tailored and reflect the specific ecological and genetic conditions of each population. This is particularly urgent given the widespread impact of overfishing on elasmobranch populations in both the United States and Brazil, which has contributed to significant declines in biodiversity and species abundance in recent decades (Sala et al. 2004; Marceniuk et al. 2019; Dulvy et al. 2021, 2024).

### 4.1 Cryptic Diversity and Taxonomic Complexity in the *Squalus* cf. *mitsukurii*

The findings of this study reinforce growing genomic evidence indicating that *Squalus* cf. *mitsukurii* does not represent a single, panmictic species, but rather a species complex composed of multiple, morphologically similar but genetically distinct lineages. This hypothesis is strongly supported by recent molecular taxonomic studies that employed DNA barcoding and species delimitation approaches. In particular, analyses using the General Mixed Yule Coalescent (GMYC) and Poisson Tree Process (PTP) models, commonly applied to mitochondrial COI datasets, have identified as many as six distinct molecular operational taxonomic units (MOTUs) within specimens morphologically assigned to *S. mitsukurii* (Ariza et al. 2022).

These results reflect considerable intraspecific genetic divergence, which exceeds typical thresholds for species delimitation in elasmobranchs and suggests the presence of unrecognized, cryptic species within the complex. In some cases, MOTUs assigned to *Squalus* cf. *mitsukurii* have been found to overlap with those attributed to *Squalus clarkae*, further blurring the taxonomic boundaries between these nominal species (Ziadi‐Künzli et al. 2020). This overlap highlights one of the key challenges in the taxonomy of *Squalus*: found clear distinction between the morphological characters (Viana et al. 2016; Veríssimo et al. 2017; Pfleger et al. 2018; Ferrari et al. 2021; Ariza et al. 2022). Members of the genus often exhibit conserved external morphology, which limits the diagnostic power of traditional morphometric and meristic traits and increases the likelihood of misidentification, both in field collections and in reference databases such as GenBank and BOLD. Consequently, many specimens labeled as *Squalus* cf. *mitsukurii* in repositories may in fact represent distinct, yet undescribed taxa. This taxonomic uncertainty not only complicates biodiversity assessments but also hampers conservation efforts, as species‐specific data on distribution, population trends, and ecological requirements become conflated or obscured (Bellodi et al. 2018; Daly‐Engel et al. 2018; Ferrari et al. 2021).

Our results contribute directly to this body of evidence and further support the hypothesis that *Squalus* cf. *mitsukurii* occurs along the Brazilian coast, although morphological similarity with other congeners makes its identification based on external characters alone highly unreliable. Supporting this, Andreoli et al. (2025) demonstrated that individuals identified morphologically as *Squalus lobularis* in the Southwest Atlantic shared mitochondrial COI haplotypes and exhibited extremely low genetic divergence (K2P = 0.35%) with *S. mitsukurii* , suggesting these names likely refer to the same species. This finding confirms the presence of *Squalus* cf. *mitsukurii* in the Argentine‐Uruguayan Common Fishing Zone (ZCPAU) and, by ecological extension, along the Brazilian continental margin, given the oceanographic continuity between these areas.

This taxonomic ambiguity carries critical conservation implications. If the Brazilian population currently attributed to *S. lobularis* is genetically identical to *S. mitsukurii* , then conservation status assessments, ecological studies, and management strategies may be misdirected (Andreoli et al. 2025). Accordingly, we strongly recommend incorporating molecular tools to validate morphological identification, ensuring greater taxonomic precision in future biodiversity and fisheries assessments.

The present study also reveals the existence of two genetically distinct stocks of *Squalus* cf. *mitsukurii*, one from California (USA) in the Pacific Ocean and the other from Pernambuco (Brazil) in the Atlantic Ocean. The analysis revealed a marked genetic differentiation between these populations, suggesting restricted gene flow across these ocean basins. This level of genetic divergence provides further support for the hypothesis that *Squalus* cf. *mitsukurii* comprises multiple genetically distinct lineages and underscores the need for taxonomic revision using integrative frameworks that combine genomic and morphological datasets.

Furthermore, the observed population structure highlights the importance of implementing region‐specific conservation actions and fishery management policies, in alignment with national regulations and international conservation priorities. Future studies should aim to identify additional genetic stocks across the species' range, conduct connectivity analyses to assess gene flow and population dynamics, and refine species delimitation. These efforts are particularly urgent given the Endangered status of *Squalus* cf. *mitsukurii* on the IUCN Red List (2025), which emphasizes the critical need for evidence‐based conservation and taxonomic clarity in this ecologically vulnerable group.

## Author Contributions

**Aisni Mayumi Corrêa de Lima Adachi:** formal analysis, methodology, validation, investigation, writing – original draft, writing – review and editing. **Ailton Amarante Ariza:** formal analysis, methodology, investigation, writing – original draft, writing – review and editing. **Giovana da Silva Ribeiro:** formal analysis, investigation, writing – original draft, writing – review and editing. **Pollyana Christine Gomes Roque:** investigation, resources, writing – original draft preparation, writing – review and editing. **Fabio Hissa Vieira Hazin:** conceptualization, investigation, resources. **Matheus Marcos Rotundo:** conceptualization, investigation, resources, writing – original draft preparation, writing – review and editing. **Marcelo Vianna:** conceptualization, investigation, resources, writing – original draft, writing – review and editing. **Gabriela Delpiani:** resources, writing – original draft, writing – review and editing. **Sergio Matias Delpiani:** resources, writing – original draft, writing – review and editing. **Juan Martín Díaz de Astarloa:** resources, writing – original draft, writing – review and editing. **Claudio Oliveira:** resources, conceptualization, writing – original draft, writing – review and editing. **Fausto Foresti:** resources, conceptualization, writing – original draft, writing – review and editing, funding acquisition. **Vanessa Paes da Cruz:** conceptualization, validation, formal analysis, investigation, resources, writing – original draft, writing – review and editing, supervision, project administration. All authors have read and agreed to the published version of the manuscript.

## Funding

This work was supported by Coordenação de Aperfeiçoamento de Pessoal de Nível Superior (88887.675590/2022‐00, 101813/2024‐4).

## Conflicts of Interest

The authors declare no conflicts of interest.

## Data Availability

I confirm that all data supporting this study will be made publicly available in an appropriate repository according to the Animal Genetics data sharing policy.
